# Laryngeal stenosis as a late complication of plasma cell mucositis of the larynx

**DOI:** 10.1093/jscr/rjaf512

**Published:** 2025-07-28

**Authors:** Gerard P Sexton, Katherine Griffin, Rachel Lockhart, Liam Skinner

**Affiliations:** Department of Otolaryngology, Head & Neck Surgery, University Hospital Waterford, Waterford X91ER8E, Ireland; Department of Otolaryngology, Head & Neck Surgery, University Hospital Waterford, Waterford X91ER8E, Ireland; Department of Histopathology, Tallaght University Hospital, Waterford D24NR0A, Ireland; Department of Otolaryngology, Head & Neck Surgery, University Hospital Waterford, Waterford X91ER8E, Ireland

**Keywords:** plasma cell mucositis, mucous membrane plasmacytosis, IgG-4 related systemic disease, tracheostomy, supraglottic stenosis

## Abstract

Plasma cell mucositis (PCM) is a rare inflammatory condition of the upper aerodigestive tract with a heterogeneous clinical course. Predominately seen in the oral cavity, laryngeal involvement and the requirement for tracheostomy have been rarely described. We describe the case of a man diagnosed with airway-threatening PCM isolated to the larynx requiring a tracheostomy. He responded to oral glucocorticoids and was decannulated only to present 3 years later with severe laryngeal stenosis requiring repeat tracheostomy insertion. Medical management in the form of immunosuppression is the mainstay of treatment for plasma cell mucositis. Surgical management is necessary in a significant proportion of cases with laryngeal involvement.

## Introduction

Plasma cell mucositis (PCM) is a chronic, non-neoplastic, inflammatory disorder of unknown aetiology defined by submucosal plasma cell infiltrates and epithelial hyperplasia [1]. The clinical presentation can involve any mucosal site, though the oral cavity is the most common [2]. The clinical course varies based on the location and degree of involved mucosa [3]. The natural history is unclear, with the literature base entirely composed of case series and reports [4].

A recent review found 23 cases of laryngeal PCM [5]. Isolated laryngeal involvement has been reported in seven cases [5–11]. Tracheostomy has been described in four cases, two of whom were successfully decannulated [9, 12]. There remains a paucity of long-term data regarding the prevalence of late complications to guide the need for long-term medical management. We describe an acute presentation of PCM with later progression to laryngeal stenosis requiring emergent tracheostomy.

## Case report

A 48-year-old Irish man presented in acute respiratory distress to the emergency department (ED). He described worsening stridor, shortness of breath, neck tightness, and voice change over the preceding days. Past medical history was notable for gout, gastro-oesophageal reflux disease, laparotomy for acute bowel ischaemia, two incisional hernia repairs, and appendicectomy. He was an ex-smoker of 20 cigarettes/day for 10 years and took minimal alcohol.

**Figure 1:**
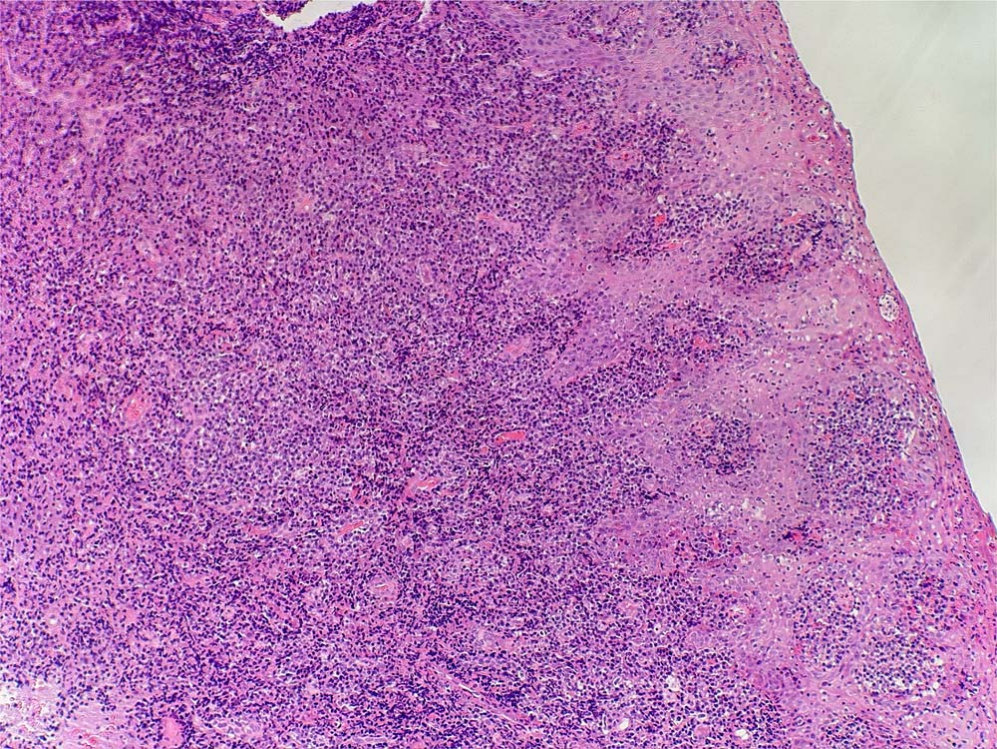
Slide from epiglottis specimen at index presentation showing squamous mucosa and underlying polyclonal plasmocytic infiltrate.

**Figure 2:**
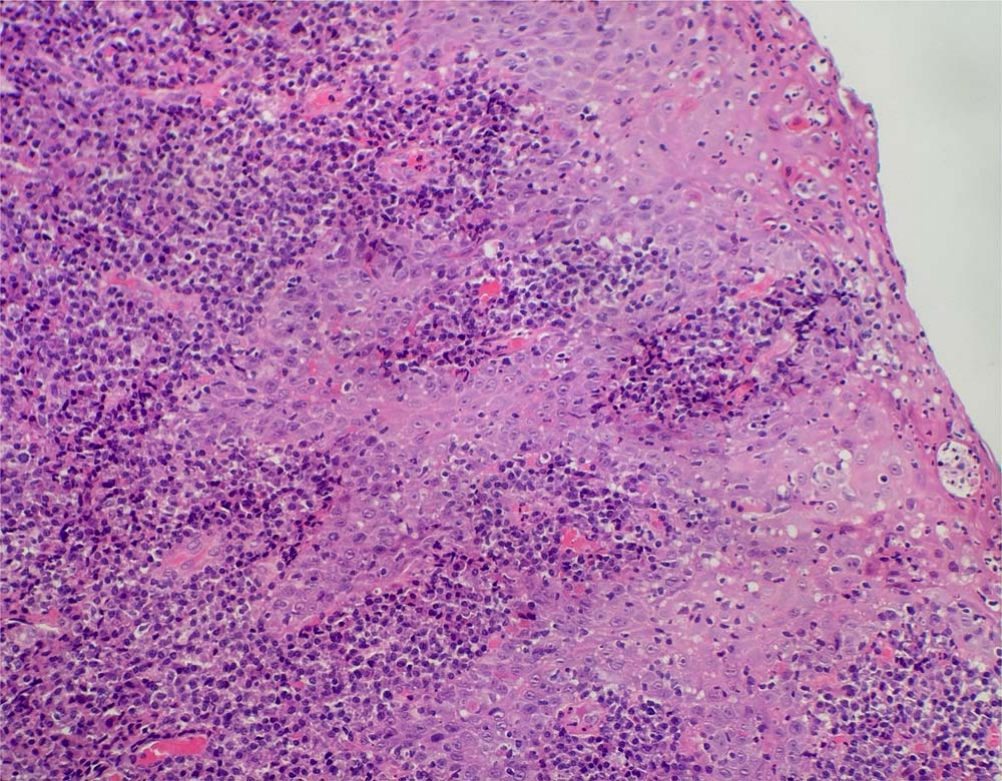
Slide from index presentation under higher magnification again demonstrating plasmocytic infiltration.

On clinical examination, harsh stridor with increased work of breathing was noted. Flexible nasoendoscopy demonstrated generalized supraglottic oedema with impending upper airway obstruction. Intravenous dexamethasone was commenced. A surgical tracheostomy with laryngeal biopsies under general anaesthetic was carried out. Histological examination (Figs 1 and 2) demonstrated hyperplastic, hyperkeratotic squamous mucosa with reactive atypia and an underlying dense polyclonal plasmocytic inflammatory infiltrate. No granulomata, prominent eosinophils, or stigmata of vasculitis were seen. Connective tissue disease screening demonstrated normal antinuclear antibody levels with positive perinuclear anti-neutrophil cytoplasmic antibodies. Anti-proteinase 3 levels returned as 3.7 IU/ml (range 0–1.9), while antimyeloperoxidase levels were normal. Rheumatology was consulted, and a provisional diagnosis of PCM was made. Serial laryngoscopy demonstrated resolution of the oedema, with the supraglottis regaining a normal appearance despite gradual tapering of the intravenous dexamethasone. A tracheostomy capping trial was successful. The patient was decannulated uneventfully and discharged on oral prednisolone. Three months later, he was maintained on 5 mg prednisolone—attempts to taper any further caused symptoms recurrence. Outpatient referral was made to Rheumatology for medical management with steroid-sparing therapy. Unfortunately, the patient was lost to follow-up due to failure to attend for outpatient review.

Three years later, the patient presented via ED with stridor and worsening shortness of breath. In the interim, he had tapered off steroids successfully but would frequently experience mild, episodic stridor requiring short courses of oral steroids. Worsening of stridor on the day led him to present via the ED.

**Figure 3:**
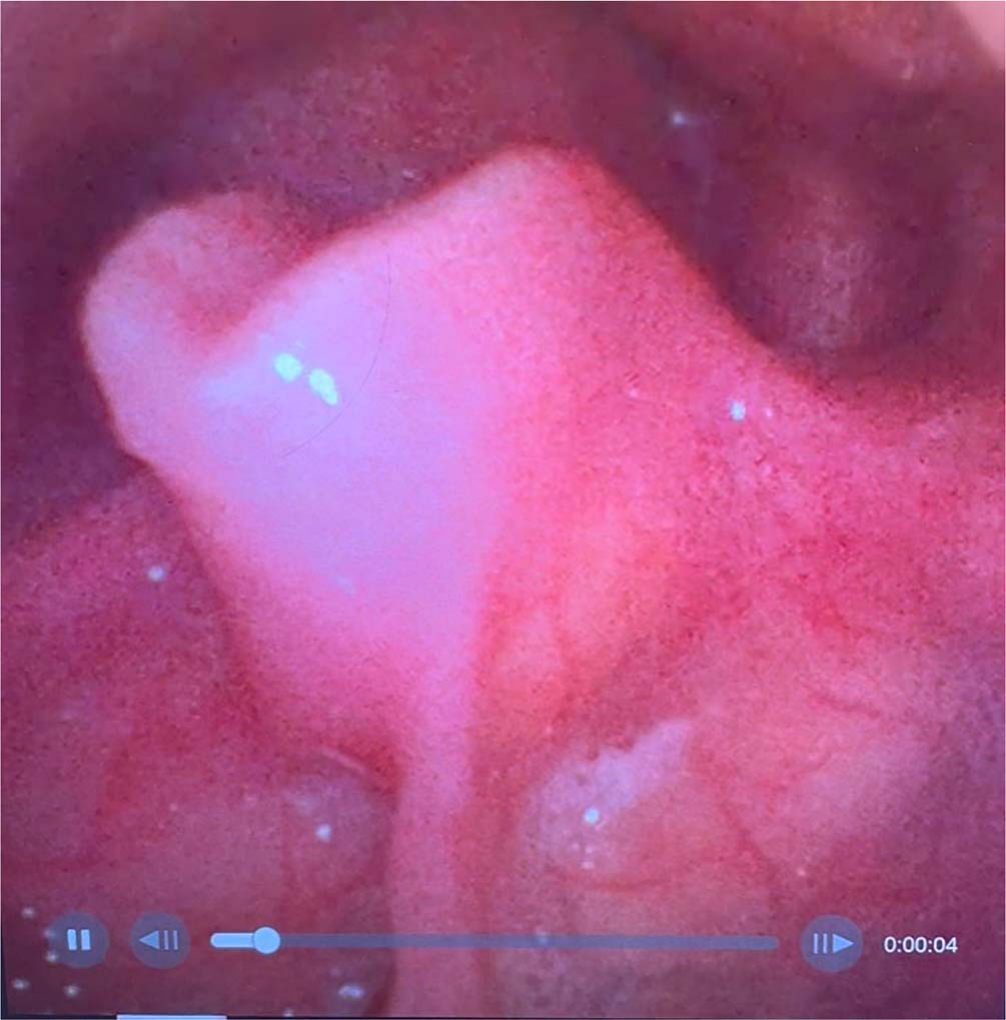
Flexible endoscopic view of a retroflexed epiglottis with a fixed, stenotic appearance of the supraglottic airway.

**Figure 4:**
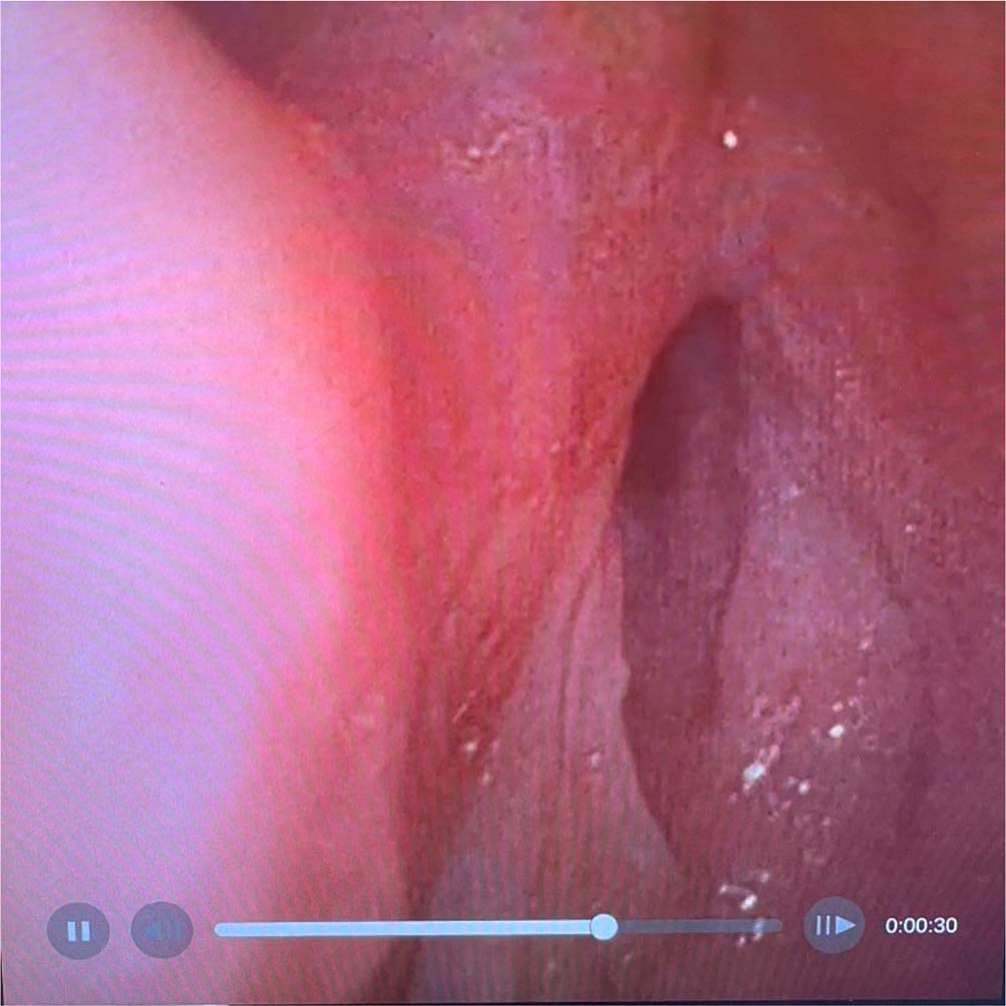
Flexible endoscopic view of best image achieved of glottic airway demonstrating severe stenosis and slit-like airway with minimal mucosal oedema.

On examination, the patient was in severe respiratory distress with harsh stridor and tripod stance. Flexible nasoendoscopy yielded images shown in Figs 3 and 4. Figure 3 demonstrates a retroflexed epiglottis which did not extend during respiration. Figure 4 demonstrates minimal cobblestoning of the mucosa and severe stenosis of the glottis. A CT thorax incidentally performed 2 weeks previously to monitor a lung nodule did not demonstrate any distal tracheal stenosis but did not capture the supraglottis. Further imaging was not undertaken in the acute phase.

The overall impression was of supraglottic/glottic stenosis with a tenuous airway in an otherwise healthy man. A tracheostomy was inserted under local anaesthesia through dense fibrotic tissue.

Subsequent immunological workup yielded similar results to the initial presentation and was negative for myeloma, IgG4-related systemic disease (IG4SD), or any underlying cause of plasmocytic infiltration. Referral for rheumatology review was again made. Given the isolated disease and the risks of immunosuppression, the consensus was for clinical surveillance with steroid-sparing therapy held in reserve. At follow-up at 9 months, the patient remains well but remains tracheostomy dependent. There has been no change in the stenotic appearance of the larynx.

## Discussion

Permanent tracheostomy likely could have been avoided with a steroid-sparing regime; tacrolimus, methotrexate, mycophenolate mofetil, and adalimumab have all been used in this setting. Pepper *et al.*’s case of a lingual squamous cell carcinoma arising from PCM illustrates one argument against immunosuppression [13]. While this does not make the case for PCM representing a premalignant entity, chronic inflammation does predispose to cutaneous malignancy in the form of Marjolin ulcers [14]. Conversely, it is known that all cutaneous malignancies occur more frequently in immunosuppressed populations. In the absence of more data, symptom burden due to PCM is likely the best indicator for immunosuppression given the risks.

Supraglottic stenosis has been described secondary to supraglottoplasty in children, blunt neck trauma, intubation injury, radiotherapy, and autoimmune conditions. Siau *et al.* presented an interesting approach to surgical airway management in a patient with IG4SD [15]. Following recurrent stenosis after balloon dilation, Siau *et al.* undertook multimodal surgical management followed by medical management of rituximab and prednisolone. Surgical management for PCM is infrequently described but overrepresented with laryngeal involvement. Similarly, 20 cases of laryngeal IG4SD are reported, 13 of which needed surgical management in some form. Stepwise surgical management based on symptomatology and involved mucosa is currently the standard of care.

## Conclusion

PCM is a rare diagnosis within the upper aerodigestive tract. Clinical suspicion and histological confirmation are required for diagnosis. Management is guided by the degree and location of the involved mucosa. Aggressive medical management is warranted with progression to surgical management following the identification of progressive or airway-threatening disease.

## Data Availability

Available at request from relevant institutions.

## References

[1] Smith ME, Crighton AJ, Chisholm DM, et al. Plasma cell mucositis: a review and case report. J Oral Pathol Med 1999;28:183–6. 10.1111/j.1600-0714.1999.tb02021.x 10235373

[2] Gilligan G, Panico R, Garola F, et al. Unusual clinical presentations of plasma cell mucositis involving oral mucosa: presentation of 2 cases and review of the literature. Oral Surg Oral Med Oral Pathol Oral Radiol 2023;136:e92–108. 10.1016/j.oooo.2023.04.010 37328328

[3] Ferreiro JA, Egorshin EV, Olsen KD, et al. Mucous membrane plasmacytosis of the upper aerodigestive tract. A clinicopathologic study. Am J Surg Pathol 1994;18:1048–53. 10.1097/00000478-199410000-00008 8092396

[4] Coppola N, Cantile T, Canfora F, et al. Pitfalls and challenges in oral plasma cell mucositis: a systematic review. J Clin Med 2022;11:6550. 10.3390/jcm11216550 PMC9659091 36362778

[5] Gillanders SL, Crotty TJ, O’Neill JP. Supraglottic mucous membrane plasmacytosis: a case report and literature review. Ann Med Surg 2025;87:335–8. 10.1097/MS9.0000000000002766 PMC11918545 40109593

[6] Smith JGF, Smith CP, Leyden PJ. Plasma cell mucositis of the larynx. Clin Case Rep 2018;6:1761–4. 10.1002/ccr3.1726 30214758 PMC6132171

[7] McLennan S, Jeffery CC. Laryngeal plasmacytosis responsive to inhaled budesonide. Laryngoscope 2023;133:70–2. 10.1002/lary.30328 35938690

[8] Makarenko VV, Vaezi AE, Brettler DB, et al. Laryngeal mucous membrane plasmacytosis with 15 year follow-up: case report and literature review. Leukem Res Rep 2020;13:100190. 10.1016/j.lrr.2019.100190 PMC6906643 31867207

[9] Mistry SG, Watson GJ, Rothera MP. Balloon dilatation to treat plasmacytosis of the supraglottic larynx. J Laryngol Otol 2012;126:1077–80. 10.1017/S0022215112001922 22906705

[10] Khan NA, McKerrow WS, Palmer TJ. Mucous membrane plasmacytosis of the upper aerodigestive tract. A case report with effective treatment. J Laryngol Otol 1997;111:293–5. 10.1017/S0022215100137132 9156074

[11] El Naderi S, Primov-Fever A, Brasnu D, et al. Isolated laryngeal plasmacytosis. Eur Ann Otorhinolaryngol Head Neck Dis 2013;130:293–5. 10.1016/j.anorl.2012.09.011 23835073

[12] Triplett J, Hee G, McLean-Tooke A, Lucas M. Long-term control of laryngeal plasma cell mucositis with systemic immunosuppression. BMJ Case Rep 2018;2018:e221333. 10.1136/bcr-2017-221333 PMC6020867 29930164

[13] Pepper T, Shekar K, Singh M, et al. Squamous cell carcinoma arising in mucosal plasmacytosis. Br J Oral Maxillofac Surg 2010;48:208–10. 10.1016/j.bjoms.2009.10.030 20045232

[14] Shah M, Crane JS. Marjolin Ulcer. StatPearls. Treasure Island (FL): StatPearls Publishing Copyright © 2025, StatPearls Publishing LLC, 2025.

[15] Siau R, Zammit M, Harper J, et al. A novel treatment for supraglottic stenosis secondary to immunoglobulin G4-related disease. Ann R Coll Surg Engl 2022;104:e133–6. 10.1308/rcsann.2021.0198 34939850 PMC10335283

